# HPV infection and bacterial microbiota in breast milk and infant oral mucosa

**DOI:** 10.1371/journal.pone.0207016

**Published:** 2018-11-05

**Authors:** Heidi Tuominen, Samuli Rautava, Maria Carmen Collado, Stina Syrjänen, Jaana Rautava

**Affiliations:** 1 Department of Oral Pathology and Oral Radiology, Institute of Dentistry, Faculty of Medicine, University of Turku, Turku, Finland; 2 Department of Paediatrics, University of Turku & Turku University Hospital, Turku, Finland; 3 Department of Biotechnology, Institute of Agrochemistry and Food Science, Spanish National Research Council (IATA-CSIC), Valencia, Spain; 4 Department of Pathology, Turku University Hospital, Turku, Finland; Universidad de Chile, CHILE

## Abstract

**Objective:**

We investigated the association between bacterial microbiota in breast milk and the infant mouth. The influence of human papilloma virus (HPV) infection on infant oral microbiota was also assessed.

**Material and methods:**

Altogether 35 breast milk and 35 infant oral samples with known HPV status were selected from the Finnish Family HPV Study cohort. In total, there were 31 mother-infant pairs. The microbiota composition was characterized by 16S rRNA gene sequencing (V3-V4 region).

**Results:**

HPV DNA was present in 8.6% (3/35) of the breast milk and 40% (14/35) of the infant oral samples. Eight shared genera between breast milk and infant oral were found; these included *Streptococcus*, *Staphylococcus*, *Unclassified Gemellaceae*, *Rothia*, *Veillonella*, *Haemophilus*, *Propionibacterium* and *Corynebacterium*. HPV status was not associated with either microbiota richness or diversity in the infant mouth. However, the infant oral microbiota clustered in different groups according to HPV status. We detected higher abundance of *Veillonella dispar* (p = 0.048) at species level in HPV negative infant oral samples. We did not detect differences in the breast milk microbiota composition related to HPV infection due to only three HPV positive milk samples.

**Conclusions:**

HPV infection is associated with distinct oral bacterial microbiota composition in infants. The direction of causality underlying the phenomenon remains unclear.

## Introduction

The role of the bacterial microbiota in human health and disease has attracted considerable research interest during the last two decades. Much less is known about the interaction between viruses and the indigenous bacterial microbiota. Human papillomavirus (HPV) is a carcinogenic virus which has been causally associated with carcinomas in both the anogenital[1] and the head and neck region[2]. In order to understand oral HPV-related carcinogenesis, there is a need to understand the natural history of HPV infection.

Breast milk is colonized with bacteria. Despite high intra- and interindividual variability, each mother harbors a unique and personalized microbial profile in her breast milk. The most predominant bacteria in human milk include *Streptococcaceae* and *Staphylococcaceae* groups followed by *Proteobacteria*, *Pseudomonadaceae*, *Bifidobacteriaceae*[3–7]. The presence of HPV has been found in breast milk in 2.5–28.8% of the samples[8–11]. However, Mammas[8] et al. (2011) were not able to detect any high-risk HPV types (HR-HPV, including HPV16, 18, 26, 31, 33, 35, 39, 45, 51, 52, 53, 56, 58, 59, 66, 68, 73 and 82) in breast milk samples whereas Sarkola[9] et al. (2008) and Yoshida[10] et al. (2011) both found HPV16 in 4.0% and 2.5%, of the studied milk samples, respectively. Interestingly, the presence of HPV DNA in mother’s milk is not a risk factor for oral HPV infection in the infant[8,10,11].

HPV has been reported to be detectable in 3.2–21% of infant oral mucosa samples[12–16]. Specific cell-mediated immunity against HPV16 has also been detected in sexually naïve infants [17]. Vertical transmission of HPV from the mother has been reported to occur prenatally via the placenta and during vaginal delivery[15,18,19]. HPV infection has also been detected in conjunctival, pharyngeal and genital sites of infants[16]. The reported maternal risk factors for vertical HPV transmission include persistent cervical HR-HPV infection, infection with multiple HPV genotypes and HPV positive placenta as well as hand warts, young maternal age at the onset of sexual activity and oral contraceptive use[12,14,15,20,21]. In addition, cord blood positive for HPV increases the risk of HPV infection in the infant[21]. In contrast, the mode of delivery has not been a predictor for vertical transmission in most studies[13]. In addition to acquiring HPV from the mother, HPV infections can also be transmitted horizontally since we have previously shown that persistent infant oral HPV infections are associated with oral carriage of HR-HPV in both the mother and the father[14,20].

The infant oral microbiota is delicate and primarily influenced by the mothers’ or care givers’ own microbiota composition particularly in breast milk, the mammary areola and in the oral cavity[22]. The development of the infant oral cavity microbiota begins directly after delivery. The first colonizers, studied from 10 minutes to 53 hours after birth, include *Staphylococcus epidermidis*, followed by *Staphylococcus aureus*, *Streptococcus mitis* and *Streptococcus oralis*[23,24]. During the first year of life the levels of anaerobic bacteria increase steadily: in two-month-old infants, the most abundant anaerobic bacteria are *Veillonella* spp. and early colonizers also include *Prevotella melaninogenica*. *Prevotella intermedia* is a late colonizer and already at one year of age the primary anaerobic species has been reported to be *Fusobacterium nucleatum*[25].

We have previously shown that the presence of HPV infection has an effect on microbiota composition in the placenta, cervix and in the oral cavity in women[26]. In this study, we extended the research to mother’s breast milk and their infant’s oral microbiota. Our aim was to investigate the similarities in the overall microbiota profile between the breast milk and the infant mouth and the association of HPV infection with these two microbiota profiles.

## Methods

The subjects and samples of the present study are a sub-set from the Finnish Family HPV Study collected during the years 1998–2001[14,20]. The original study was designed to evaluate the interactions of HPV infection within families. Altogether 329 women were enrolled to this study in their final trimester, together with their male spouses (n = 131) and offspring to come (n = 313) with a 6-year follow-up period. Written informed consent was obtained from all participants and the study design was found acceptable by the Ethics Committee of the Intermunicipal Hospital District of Southwest Finland (#3/1998, #2/2006, 45/180/2010).

We selected a total of 40 infant oral and 39 breast milk samples from the Finnish Family HPV study from our previously published study population described in detail by Tuominen et al. (2018)[26]. Because some of the originally selected samples had low DNA quality for further analyses, we were forced to exclude 5 infant oral and 4 breast milk samples from microbiota analyses. Therefore, the final study population consisted of 35 infant oral and 35 breast milk samples. Because not all of the excluded samples were from the same mother-infant pairs, we ended up with 31 paired samples. The samples were collected at two different time points due to family-related reasons. Thirteen infant oral samples were collected at birth and 22 samples at the age of two months. Altogether, 31 breast milk samples were obtained at birth and four of them two months postpartum.

### HPV detection and genotype

HPV DNA was extracted by the high-salt method as described in Miller[27] et al. (1988). HPV genotyping was performed by Multimetrix (Progen Biotechnik GmbH, Heidelberg, Germany) as described in Koskimaa[28] et al.(2012) and Louvanto[11] et al. (2017). The HPV genotypes of all of the samples has been analyzed and reported earlier from infant oral[28] and from breast milk[11].

### 16S bacterial gene sequencing and analysis

The sample analyzing have previously been described in detail[26]. Briefly, the V3-V4 region of the 16S rDNA gene was amplified following Illumina protocols using Nextera XT Index Kit (Illumina, San Diego, CA, USA). Five infant oral samples and four breast milk samples were excluded from the analyses due to the poor quality of the DNA. Thus, the final cohort comprised of 35 breast milk and 35 infant’s oral samples. The libraries were sequenced using a 2x300pb paired-end run (MiSeq Reagent kit v3) on a MiSeq-Illumina platform (Lifesequencing sequencing service, Valencia, Spain). The PCR amplification and library controls were sequenced along as controls to exclude possible contaminations.

After primer removal, sequences with <300 nucleotides read length were trimmed and quality filtered. Chimeric sequences were filtered using UCHIME program version 4.2. An open reference OTU picking method using 99% identity to the Greengenes 13_8 database was performed using QIIME pipeline (version 1.9.0)[29]. Singletons and OTUs with a relative frequency below 0.01 were removed. Sequences, that could not be classified to domain level, or were classified as *Cyanobacteria*, *Chloroplasts* and *Rhizobiales* were removed.

Alpha diversity indices (Chao1 index: richness and Shannon index: diversity) and beta diversity using UNIFRAC (phylogenetic) and Bray Curtis distance (non-phylogenetic) were obtained. The Analysis of similarities (ANOSIM) test was used to test for statistically significant differences between the groups. Calypso software version 8.24 (http://cgenome.net/calypso/) was used with total sum normalization (TSS) for the statistical analysis. Linear discriminant analysis effect size (LEfSe) was used to detect unique biomarkers (linear discriminant analysis (LDA) score>3.0) in relative abundance of bacterial taxonomy. Inter and intraindividual variability was identified by pair-level analyses (paired t-test). We considered p-values ≤0.05 as statistically significant.

## Results

### HPV status of the samples

The HPV status of the complete Finnish family HPV study samples has been previously reported by Koskimaa[28] et al. (2012) and Louvanto[11] et al. (2017). In the present sub cohort of 31 mother-infant pairs, HPV DNA was detected in 8.6% of the mother’s breast milk and 40% of the infant oral samples. The genotypic distribution of all of these samples is presented in Table 1. The study subjects included one set of twins. Infection with multiple HPV types was found in one infant oral sample and in none of the milk samples. Table 2 depicts the HPV status according to the sex of the infant, delivery mode and duration of gestation within the mother-infant pairs.

**Table 1:** HPV genotype distribution among samples.

HPV genotype/s	Infant oral (one set of twins) n = 14/35 (40%)	Mother’s breast milk n = 3/35 (8,6%)
HPV 16	6/35 (17%)	1/35 = 3%
HPV 6	1/35 (3%)	2/35 = 6%
HPV 18	2/35 (6%)	
HPV 66	2/35 (6%)	
HPV 70	1/35 (3%)	
HPV 58	1/35 (3%)*	
multiple types (HPV 33 and 59)	1/35 (3%)	

*mother had HPV 6 in her breast milk, other breast milk HPV positive mothers had HPV negative infants

**Table 2:** HPV status of the samples.

ID	Infant sex	Delivery mode (VD vaginal delivery, CS caesarean section)	Gestation weeks	Infant oral (HPV type)	Timepoint when infant oral samples were collected	Mother’s milk (HPV type)	Timepoint when breast milk samples were collected
1	female	VD	42.0	(-)	at 2 months		at birth
2	female	VD	41.0	33,59	at birth		at birth
3	female	CS	35.4	(-)	at birth		at birth
4	female	VD	40.1		at 2 months		at birth
5	male	VD	37.6		at 2 months		at 2 months
6	female	VD	40.1		at 2 months		at birth
7	male	VD	42.0		at 2 months		at birth
8	female	VD	39.4		at 2 months		at birth
9	female	VD	38.3		at 2 months	(-)	at birth
10*	male	VD	38.0		at 2 months		at birth
11*	male	VD	38.0	18	at 2 months		at birth
12	male	CS	38.5		at 2 months		at birth
13	male	CS	42.2		at 2 months		at birth
14	female	VD	41.5	18	at 2 months		at birth
15	male	VD	41.0		at 2 months		at birth
16	male	VD	39.0		at birth		at birth
17	female	CS	40.0	16	at birth		at birth
18	male	CS	42.1		at 2 months		at birth
19	female	CS	42.4	6	at 2 months		at birth
20	female	CS	41.1		at 2 months		at birth
21	female	VD	39.6		at 2 months		at birth
22	male	CS	38.4	66	at birth	(-)	at birth
23	female	VD	39.0	16,33(-)	at birth		at birth
24	male	VD	42.0		at birth	16	at birth
25	female	VD	40.4	16	at birth		at birth
26	female	CS	39.0		at 2 months		at birth
27	male	VD	40.4	16	at birth		at birth
28	female	VD	41.0	70	at 2 months		at birth
29	female	CS	39.1		at 2 months		at birth
30	female	VD	41.6	16	at 2 months		at birth
31	female	CS	40.6		at 2 months		at birth
32	male	VD	38.3	66	at birth		at 2 months
33	female	VD	40.6		at 2 months		at 2 months
34	female	VD	40.0	58	at birth	6	at birth
35	female	CS	39.0		at birth	6	at birth
36	female	CS	41.4		at birth	(-)	at birth
37	male	VD	39.6	6(-)	at birth		at birth
38	male	CS	35.3	16	at birth	(-)	at birth
39	male	VD	36.6	6(-)	at birth		at birth
40	male	CS	42.0	16	at birth		at 2 months

*twins

(-) samples not included in the analyses because of the poor DNA quality

### Bacterial microbiota composition in breast milk and the infant oral cavity

**Fig 1:**
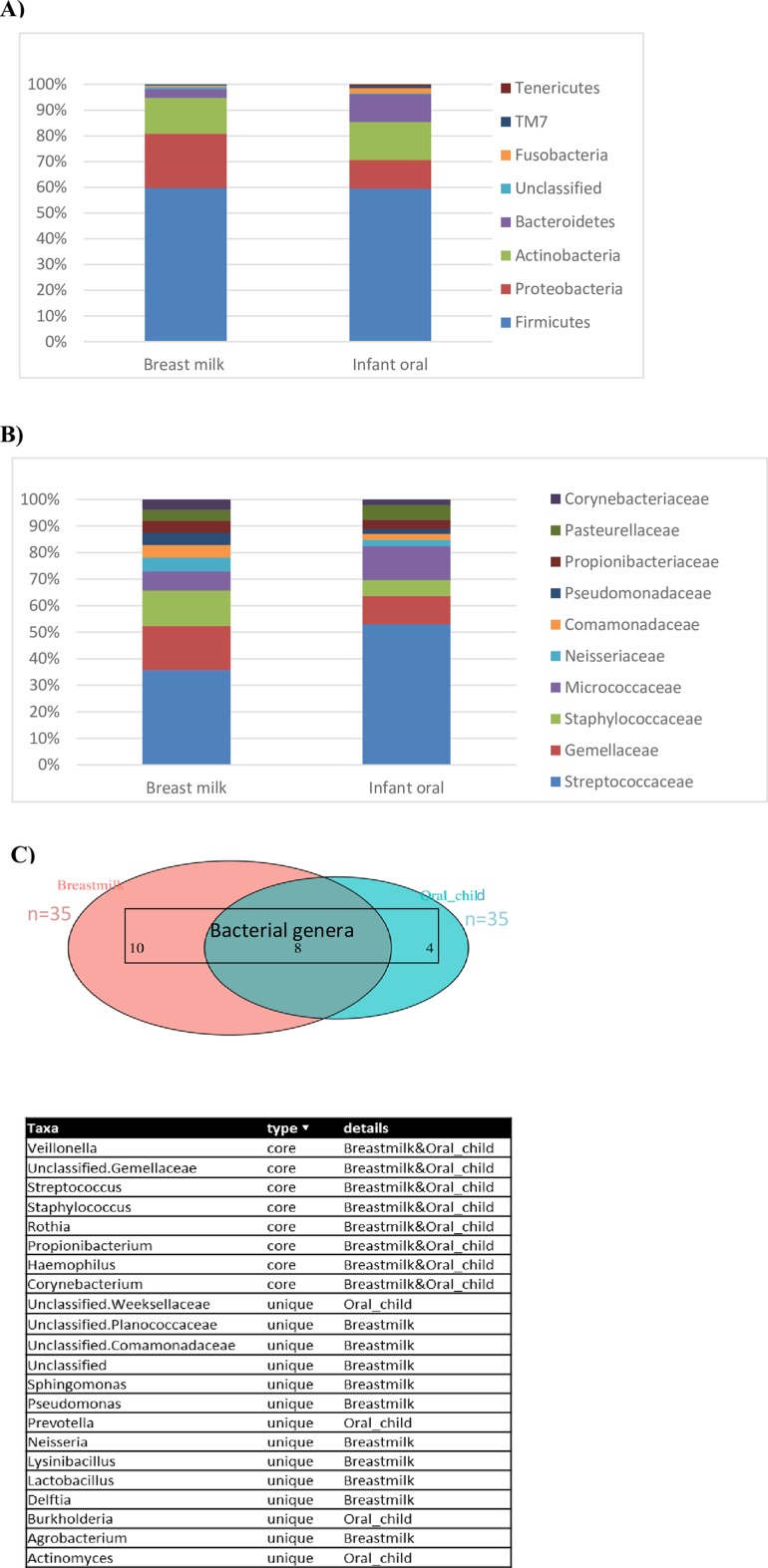
Bacterial microbiota composition in breast milk and the infant oral cavity. The relative abundance of bacteria are presented on the phylum (Fig 1A) and family levels (Fig 1B) for both groups. A Venn diagram with the list of shared and unique bacterial genera both in breast milk and infant oral samples (Fig 1C).

The most abundant phyla in breast milk and infant oral cavity were *Firmicutes* (79.7% vs 82.6%, respectively), *Proteobacteria* (14.1% vs 4.5%, p = 0.0082), *Actinobacteria* (5.2% vs 6.4%) and *Bacteroides* (0.5% vs 6.0%, p = 0.009) (Fig 1A). The predominant microbial families belonged to *Streptococcaceae* (50.7% vs 59.4%), *Gemellaceae* (15.3% vs 4.2%, p = 0.0012), *Staphylococcaeae* (11% vs 3.9%, p = 0.0078) (Fig 1B). A core of eight genera was observed between breastmilk and infant oral cavity as shown by Venn diagram (Fig 1C). This core microbiota was composed of *Streptococcus*, *Staphylococcus*, *Unclassified Gemellaceae*, *Rothia*, *Veillonella*, *Haemophilus*, *Propionibacterium* and *Corynebacterium*.

### The effect of age on infant oral microbiota

**Fig 2:**
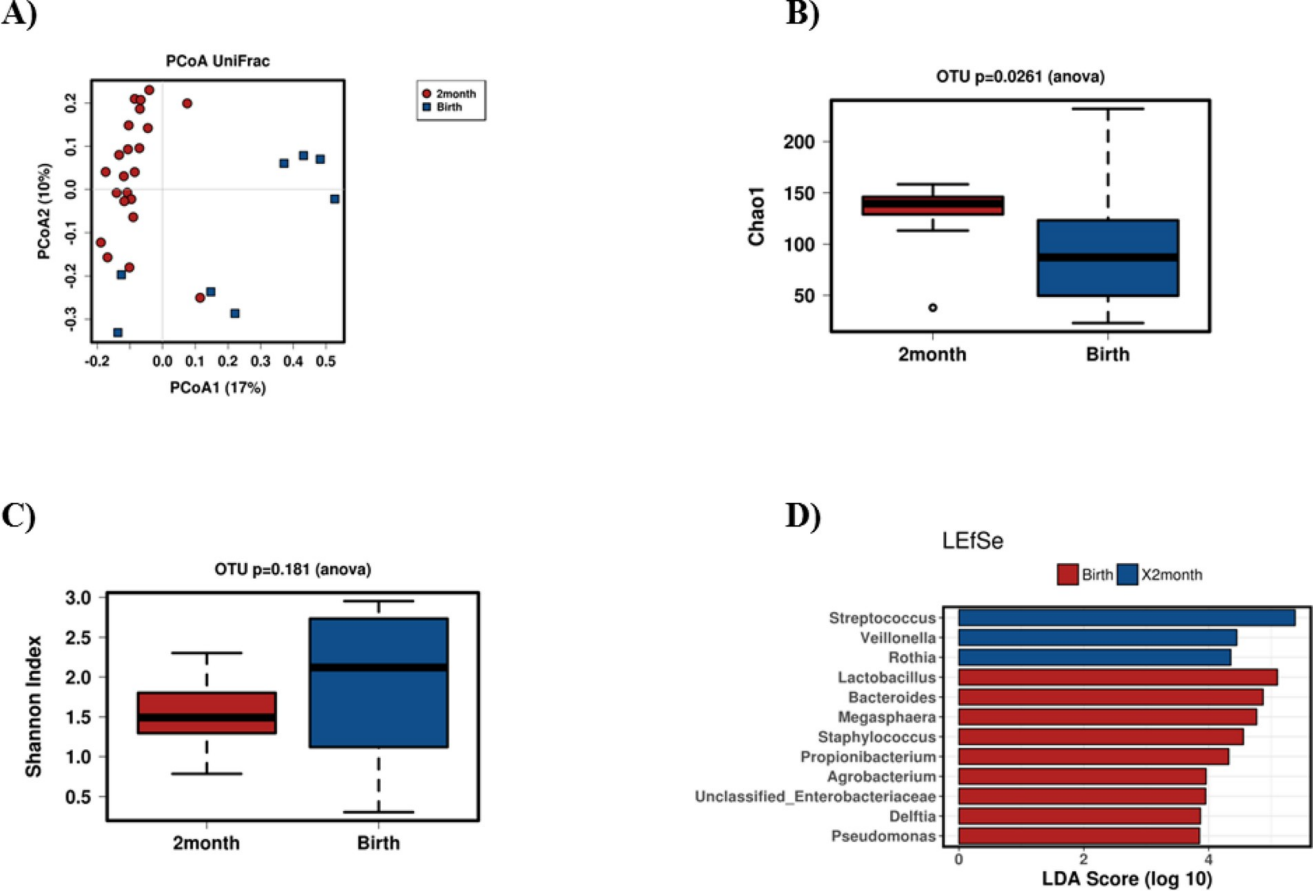
The effect of age on infant oral microbiota. PCoA chart depicting two different clusters in relation to the time (at birth = blue, at two months of age = red, Fig 2A). Higher richness (p = 0.026, Chao 1 index; Fig 2B) was observed in infant oral samples collected at 2 months of age (red) versus samples collected at birth (blue) but no difference in diversity were evident (Shannon index; Fig 2C). LEfSe test showing different abundances between the two different time groups (blue = two months, red = birth, Fig 2D).

Infant age had a substantial impact on infant oral microbiota composition. Two distinct clusters in relation to age are seen when samples obtained at birth (n = 13) are compared to those collected two months (n = 22) after birth (Fig 2A). Furthermore, higher richness (p = 0.026, Chao 1 index, Fig 2B) was observed in the infant oral samples collected at two months of age when compared to infant oral samples taken at birth. No difference in diversity was detected (p = 0.18, Shannon index, Fig 2C). With LEfSe (linear discriminant analysis effect size) test, higher abundance of *Streptococcus*, *Veillonella* and *Rothia* (LDA score>4.0, p<0.05) was found in the two-month-old infant oral samples, whereas *Lactobacillus*, *Bacteroides* and *Megasphaera* (LDA score>4.0, p<0.05) were higher in the infant oral samples collected at birth (Fig 2D).

At phylum level, the levels of *Micrococcaceae* (p = 0.000051) and *Streptococcaceae* (p = 0.0051) were higher in samples taken two months after birth when compared to elevated level of *Tissierellaceae* (p = 0.033) in samples taken at birth. Furthermore, at species level *Rothia mucilaginosa* (p = 0.000043), *Unclassified Streptococcus* (p = 0.0051), *Unclassified Weeksellaceae* (p = 0.0095), *Unclassified Haemophilus* (p = 0.032) and *Veillonella dispar* (p = 0.034), *Unclassified Anaerococcus* (p = 0.030) and *Unclassified Finegoldia* (p = 0.052) levels were all higher at the age of two months compared to the samples taken at birth.

### Differences and similarities between breast milk and infant oral microbiota

**Fig 3:**
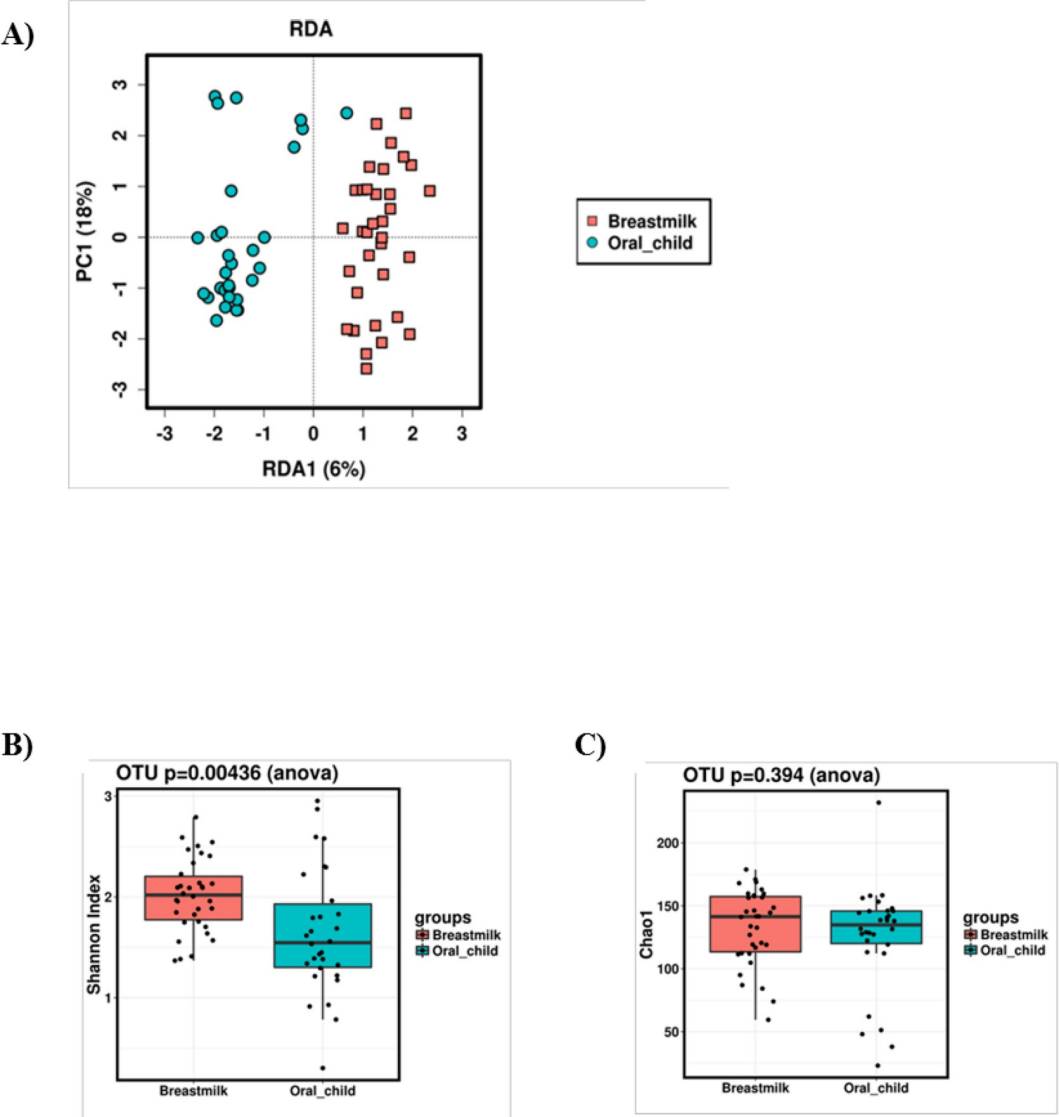
Differences and similarities between breast milk and infant oral microbiota. Multivariate redundancy discriminant analysis (RDA) showing differences between breast milk (light red) and infant oral cavity (light blue) microbiota profiles (Fig 3A). Higher diversity (p = 0.0044, Shannon index, Fig 3B) was detected in breast milk (light red) as compared to infant oral (light blue) samples, but no differences in richness were observed (Chao 1 index, Fig 3C).

Significant differences in the microbiota profile were found between breast milk and infant oral cavity using multivariate Redundant Discriminant Analysis (RDA) (p = 0.0001, Fig 3A). The comparisons were performed by clustering all mothers’ breast milk microbiome data together and compared it to the clustered infant oral microbiota data. Furthermore, breast milk showed higher microbial diversity as compared to the infant oral microbiota (p = 0.0043, Shannon index, Fig 3B) but no differences in bacterial richness (p = 0.394, Chao1 index, Fig 3C) were detected.

No statistical differences were observed in breast milk or infant oral microbiota composition according to the mode of delivery either with PCoA or with RDA (p = 0.570 and p = 0.456, respectively). When analyzing the microbiota data of the 31 mother- infant pairs we were not able to identify pairwise similarities or to connect mother to her infant by microbiota profile.

### Breast milk microbiota and HPV

**Fig 4:**
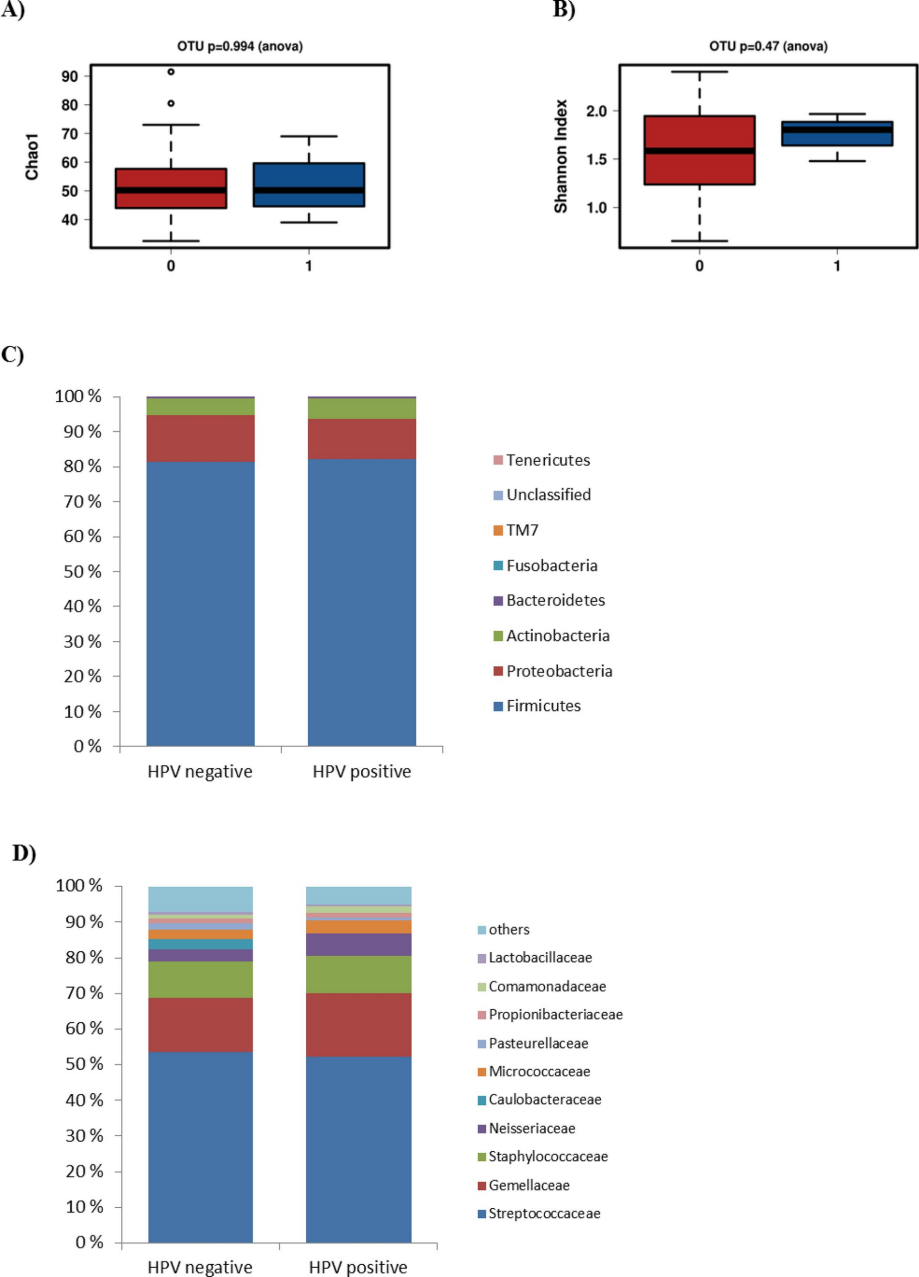
Breast milk microbiota and HPV. No differerence in richness (Chao 1 index, Fig 4A) or diversity (Shannon index, Fig 4B) were detected between HPV postitive (blue) and HPV negative (red) breast milk samples. The relative abundances of bacteria are presented on the phylum (Fig 4C) and family levels (Fig 4D).

There were only three HPV positive breast milk samples. No statistically significant differences in species richness (Fig 4A) or diversity (Fig 4B) were detected between the HPV positive and HPV negative breast milk samples, nor did we observe any statistical differences in microbial compositions at phylum or at family levels in the breast milk samples (Fig 4C and 4D).

### Infant oral HPV infection and bacterial microbiota

**Fig 5:**
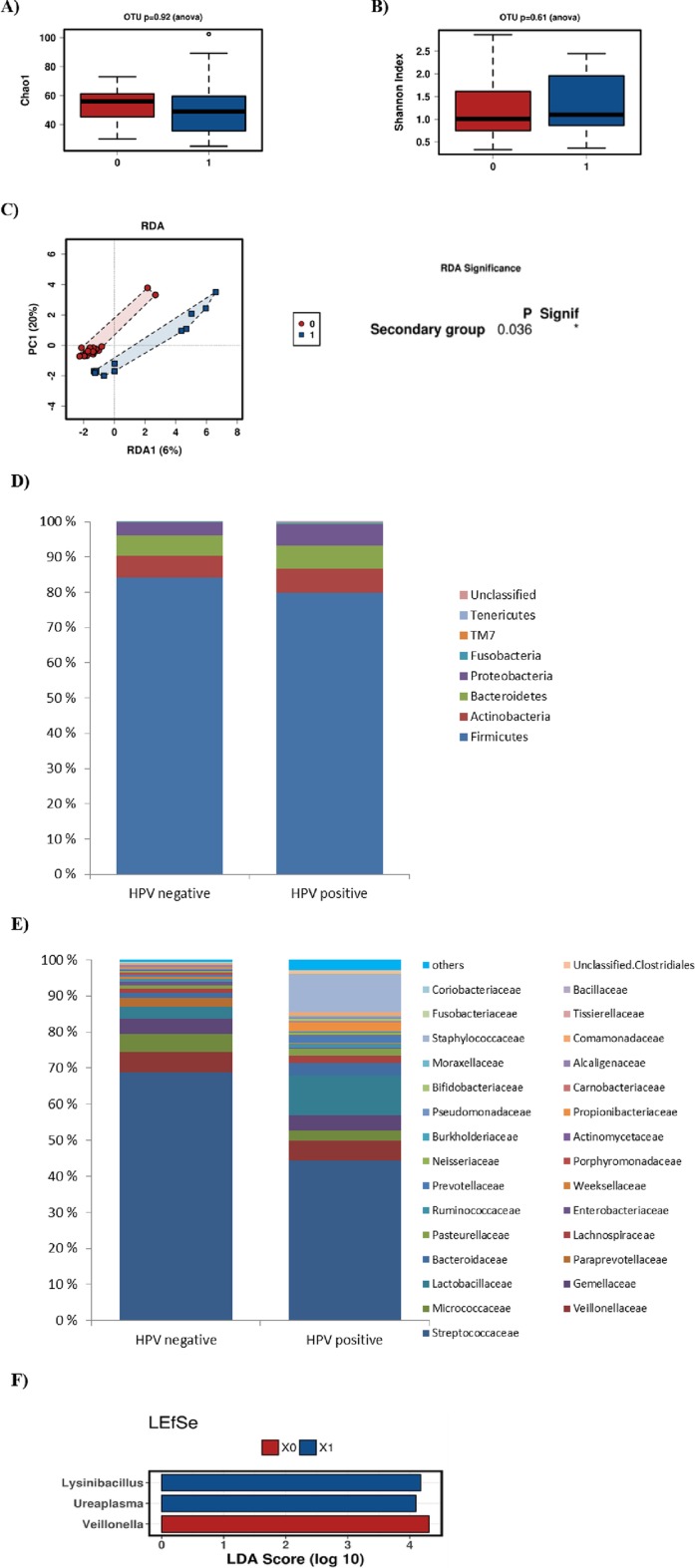
Infant oral HPV infection and bacterial microbiota. No differences in bacterial richness (Chao 1 index, Fig 5A) or diversity (Shannon index, Fig 5B) between HPV positive (blue) and HPV negative (red) infant oral samples were detected. RDA analysis (Fig 5C) shows significant differences between HPV positive (blue) and HPV negative infant oral samples (red). The relative abundances of bacteria are presented on the phylum (Fig 5D) and family levels (Fig 5E). By LEfSe analysis (Fig 5F), *Lysinibacillus* and *Ureaplasma* were significantly enriched in HPV positive (blue) infant oral samples (LDA score >4.17 and 4.10 respectively, p<0.05), while *Veillonella* (LDA score>4.31, p<0.05) were enriched in HPV negative (red) compared to HPV positive infant oral samples.

There were no differences between richness (Fig 5A) or diversity (Fig 5B) in infant oral samples grouped by the HPV status of the samples. Nonetheless, statistically significant clustering of the oral microbiota from HPV positive and negative infants was detected by RDA (p = 0.036) (Fig 5C). No statistically significant differences in microbial composition at phylum level were detected between HPV positive and HPV negative infant oral samples (Fig 5D). However, *Proprionibacteriaceae* (p = 0.095) was increased at family level, *Propionibacterium* (p = 0.095) at genus level and *Propionibacterium acnes* (p = 0.093) at species level in HPV positive infant oral samples. In the HPV negative infant oral samples, higher abundance of *Veillonella* (p = 0.025) at genus level and *Veillonella dispar* (p = 0.048) at species level was detected. (Fig 5E).

With LefSE tests, *Lysinibacillus* and *Ureaplasma* (LDA scores>4.0, p<0.05) were significantly increased in the HPV positive group compared to the HPV negative infant oral sample group, whereas *Veillonella* (LDA score>4.0, p<0.05) was higher in HPV negative infant oral samples (Fig 5F).

The impact of HPV infection on infant oral microbiota was investigated at two different time points albeit the sample size was relatively small. No differences in bacterial richness, diversity or composition were detected when comparing the infant oral HPV negative (n = 4) and the infant oral HPV positive (n = 8) samples collected at birth. However, we observed higher richness in the infant oral HPV negative samples collected two months after birth (n = 17) when comparing to the HPV positive infant oral samples at two months of age (n = 5). No differences in diversity were seen. *Veillonellaceae* (p = 0.025) and *Veillonella dispar* (p = 0.052) levels were higher in the HPV negative infant oral samples collected two months after birth when compared to the HPV positive two months old infant’s oral samples.

## Discussion

Our results suggest for the first time that HPV infection is associated with alterations in the microbiota in the infant oral cavity. The changes are more pronounced at genus and family levels, but they do not influence species richness or diversity as a whole. Nonetheless, two distinct infant oral microbiota clusters dependent on HPV status were observed with RDA analysis. In contrast, we were not able to detect differences in breast milk microbiota composition associated with HPV infection, possibly because the total number of HPV positive breast milk samples was insufficient. Nevertheless, we still performed microbiota analyses, because the number of HPV positive milk samples (8.6%) were in line with previous studies[8–10].

The infant oral microbiota observed in this study corresponds to previous studies, which indicate that the most abundant taxa in the infant oral microbiota belong to *Streptococcus*, *Veillonella*, *Neisseria* and *Firmicutes*[5,22]. The early infant oral microbiota differs from the oral microbiota at later stages of childhood, which may in part be attributable to the absence of hard dental tissues in early infancy. Our data demonstrated that the oral microbial community evolves during the first two months of extra uterine life. Previously, the changes in microbial communities have been reported to stabilize within the first few months of life[30].

While factors including prematurity, maternal intrapartum antibiotics, delivery mode and formula feeding all affect infant oral microbiota composition[30–32], the microbes from the maternal mouth as well as the mammary areola and breast milk are the primary sources of bacteria for the infant mouth[5,22]. In our study population, we detected a shared core of eight bacterial genera between breast milk and infant oral mucosa microbiota. We did not observe any correlation between infant oral microbiota and the mode of birth in oral mucosa samples obtained at birth or at two months of age despite a previous report indicating that delivery affects the infants oral microbial composition directly after birth[33].

The breast milk microbiota is predominantly composed of bacteria which are thought to contribute to the infant’s newly developing gut microbiota and favorable microbiota prevents pathogens from colonizing the infant gut[3,5,34]. The bacterial composition of breast milk and the infant oral microbiota seem to have a connection, which is quite easy to understand since breast milk is often the first food source for the infant. This was also one of the main findings in our study; we were able to recognize a core of eight bacteria which were shared between mother’s breast milk and the infant oral cavity. This core microbiota was composed of *Veillonella*, Unclassified *Gemellaceae*, *Streptococcus*, *Staphylococcus*, *Rothia*, *Haemophilus* and *Cornyebacterium*. These data are consistent with previous study by Biagi[5] et al. who concluded that passage of breast milk through infant’s mouth has an effect on the oral microbiota composition as they also found *Staphylococcus*, *Streptococcus* and unclassified *Gemellaceae* shared between infant oral mucosa and breast milk microbiota. In our study, all of the infants received breast milk, though we are not aware, if there have been any additional food supply alongside breast feeding during the time when samples were collected. Because the main food supply has been from mother’s milk, we are confident that the results present an actual interaction between infant oral and breast milk microbiota.

The genera *Propionibacterium*, *Lysinibacillus* and *Ureaplasma* were enriched in HPV positive infant oral mucosal samples when compared to HPV negative samples. *Propionibacteriumin* has been found in the gut microbiota of preterm infants[35]. Oral *Ureaplasma* has previously been linked to HPV positivity in sexually active men[36] and *Ureaplasma* found in the cervix and placenta is connected to preterm birth without the presence of HPV[37]. Recently, *Ureaplasma* has also been discovered in infant oral cavity collected 15 minutes after vaginal birth[38]. Despite the fact that *Ureaplasma* and *Propionibacterium* have previously been connected to preterm birth, our study had only total of three infants born slightly preterm and all of them were born after 35 weeks of gestation. Still it seems that HPV infection affects oral microbiota composition by favoring the growth of pathogens, which may have clinical significance later in life.

An increase in the level of oral *Veillonella* spp. was observed in HPV negative infant oral samples as compared to positive samples at two months of age. This finding is line with previous studies indicating that *Veillonella* is one of the most abundant bacteria in infant oral cavity [25]. Later in life, oral *Veillonella* has been associated with higher caries activity[39] and poor oral hygiene[40].

Breastfeeding confers several significant health benefits to the infant. In addition, breast milk serves as one of the most important sources of microbes for the infant[5,34,41]. Healthy breast milk microbiota is mainly composed of *Proteobacteria*, *Streptococcaceae*, *Pseudomonadaceae*, *Bifidobacteriaceae* and *Staphylococcaceae*[5–7], but the composition seems to be very variable with geographical differences between individuals[3,4,6]. Even delivery mode has been reported to have effect on the microbial composition of breast milk. In particular, levels of *Lactobacillus* have been reported to be higher in milk samples from mothers who delivered by caesarean section compared to vaginal delivery[6]. In our breast milk samples, however, we did not observe differences in neither of the groups regarding the microbial composition according to the birth mode, possibly because of the low number of samples studied. Even though we did take the birth mode into account and selected samples according to it, the overall numbers of vaginal delivery and caesarean section probably remained too few to reach to statistical significance between the two groups.

The Finnish family HPV study population and samples are exceptional. The study has been designed to evaluate HPV interactions within families, and consequently all of the samples have been collected within the same family. Nonetheless, our study has limitations which include the fact that only three breast milk samples were HPV DNA positive. The observed prevalence of 8.6% HPV positivity in breast milk is in line with previous studies[8–10]. Still, possibly because of the low number of HPV positive breast milk samples we failed to reach statistical significance between microbial compositions in HPV positive and HPV negative breast milk samples. Furthermore, we were forced to exclude number of infant oral and breast milk samples from further analysis, because of the poor quality of DNA or the low number of sequences (n = 5, n = 4, respectively). Only samples that reached 1000 reads were included in the analyses. This study concentrated on investigating only selected HPV genotypes (low-risk HPV including HPV6, 11, 42, 43, 44 and 70 and high-risk HPV types HPV 16, 18, 26, 31, 33, 35, 39, 45, 51, 52, 53, 56, 58, 59, 66, 68, 73, and 82) because these mucosal HPV genotypes are the most relevant to the pathogenesis of human disease according to present knowledge[42].

We have taken into consideration possible contamination and sequenced negative controls from PCR amplification and libraries. Furthermore, we aimed to verify our results by excluding singletons and OTUs with a relative frequency below 0.01 when analyzing of the data. In addition to that, sequences that were classified as *Cyanobacteria*, *Chloroplasts* and *Rhizobiales* or sequences that could not be classified to domain level were removed from the dataset.

In conclusion, present HPV infection in infant oral cavity is connected to detectable changes in microbial composition and it also seems to favor pathogens. Still, we do not know whether present HPV infection induces these changes in microbiota composition or whether the altered microbiota colonization predisposes to HPV infection. We were not able to find alterations in breast milk samples when taken into account present HPV infection. Finally, we conclude that infant oral and breast milk share similar features, as we found a core of eight shared bacteria.
